# Adherence to the Mediterranean Diet and Its Association with Cognitive Function in Children and Adolescents: A Systematic Review of Observational Studies

**DOI:** 10.3390/children12060789

**Published:** 2025-06-17

**Authors:** Guillermo García-Pérez-de-Sevilla, Rafael Zapata-Lamana

**Affiliations:** 1 Department of Physiotherapy, Faculty of Medicine, Health and Sports, European University of Madrid, Calle Tajo s/n, Villaviciosa de Odón, 28670 Madrid, Spain; 2 Escuela de Kinesiología, Facultad de Salud, Universidad Santo Tomás, Los Ángeles 4430000, Chile; rafaelzapata@udec.cl; 3 Escuela de Educación, Campus Los Ángeles, Universidad de Concepción, Los Ángeles 4440000, Chile

**Keywords:** Mediterranean diet, nutrition, cognition, children, adolescents, lifestyle

## Abstract

Objectives: This study aimed to analyze the associations between cognitive functions and adherence to the Mediterranean diet (MedDiet) in children and adolescents. Methods: A systematic review was conducted in three databases: Pubmed, Scopus, and Web of Science. The inclusion criteria of the studies were (i) population: healthy children or adolescents; (ii) exposure: adherence to the MedDiet; (iii) outcomes: cognitive performance; and (iv) study design: observational studies only. The quality of the studies was assessed through the Newcastle–Ottawa Scale. Results: This systematic review included 12 cross-sectional, observational studies including a total of 6378 children aged 4–17 years from different countries, analyzing diet characteristics and cognitive functions. The quality of the studies was high (Newcastle–Ottawa Scale mean score = 7.42). Positive associations were found between adherence to the MedDiet and multiple cognitive functions: memory, attention, creativity, language skills, and executive functions. However, the wide variety of instruments assessing the adherence to the MedDiet and cognitive functions did not allow us to perform a meta-analysis. Conclusions: The MedDiet should be further considered and promoted among children and adolescents, as it shows promise as a dietary pattern that may support cognitive development in youth.

## 1. Introduction

The Mediterranean diet (MedDiet) is the dietary pattern with the most scientific evidence on its health effects. This dietary pattern is characterized by low intake of saturated fats and high consumption of monounsaturated and polyunsaturated fatty acids, antioxidants, polyphenols, and fiber. These nutrients are highly present in fruits, vegetables, legumes, nuts, olive oil, whole grains, and fish [1,2]. Despite the many health benefits of the MedDiet, the adherence to this dietary pattern has been reduced in the Mediterranean region in the last decades [3].

Among the many beneficial health effects of the MedDiet is its positive impact on cognitive function, which can be attributed to various nutrients inherent in this dietary pattern. For example, the consumption of healthy fatty acids, vitamins C, E, and B-12, flavonoids, and carotenes has been associated with improved cognitive outcomes in adults [4]. Also, regular intake of polyphenols has been associated with lower blood pressure, improved lipid profile, and anti-inflammatory effects, all of which contribute to enhanced cognitive function [5,6]. Moreover, the high intake of omega-3 fatty acids, abundant in oily fish and nuts, increases cerebral blood flow and the electrical potential of neuronal membranes, enabling memory formation [7]. From an anatomical point of view, adherence to the MedDiet has been linked to increased volumes of white and gray brain matter [8,9]. Conversely, high consumption of refined carbohydrates dysregulates brain glucose and reduces perfusion in the prefrontal cortex, thus negatively affecting cognitive performance [10].

Cognitive function refers to the mental processes related to acquiring, storing, and utilizing knowledge. In children and adolescents, cognitive functions are crucial for learning, problem-solving, decision-making, and overall intellectual development. Several studies have observed strong positive associations between cognitive function and academic performance [11,12,13]. Some relevant domains of cognitive functions that are closely related to learning and academic achievement in children and adolescents are attention [14], memory [11,15], language [16], creativity [17], and executive functions [18]. The executive functions are basic cognitive abilities that lead to planning, flexibility, and self-regulation [19].

Cognitive function significantly develops from childhood through adolescence, depending on brain maturation, genetic predispositions, education, social interactions, physical health, and nutrition [20]. Concerning the nutritional aspect, a recent meta-analysis found that a higher adherence to the MedDiet could play a relevant role in academic performance among children and adolescents. However, given the observed weak effect sizes and moderate inconsistency in the findings, future studies should aim to explore other contextual factors that may influence the strength of the association, such as socioeconomic factors [21]. Moreover, most studies analyzing the association between adherence to this dietary pattern and cognitive function only focus on adults, and there is not a systematic review about this topic. Therefore, the objective of this study was to analyze the associations between cognitive function and adherence to the MedDiet in children and adolescents.

## 2. Methods

This systematic review followed the Preferred Reporting Items for Systematic Reviews and Meta-analyses (PRISMA) guidelines [22] and was prospectively registered in the International Prospective Register of Systematic Reviews (PROSPERO) on 16 July 2024.

### 2.1. Eligibility Criteria

To be considered eligible for inclusion in this systematic review, a PECOS (Population, Exposure/Comparator, Outcomes, and Study type) strategy was established: (i) population: children or adolescents; (ii) exposure: adherence to the MedDiet; (iii) outcomes: cognitive performance (e.g., attention, memory, language, creativity, and executive functions); (iv) study design: observational studies only, excluding gray literature (e.g., abstracts, conference proceedings, and editorials).

The exclusion criteria were the following: (i) abstract not in English; (ii) studies carried out with children or adolescents with diseases or disabilities; (iii) focused on other dietary patterns rather than the MedDiet.

### 2.2. Data Source and Search Strategy

The following databases were examined by two independent researchers (GGPS and RZL): PubMed, Scopus, and Web of Science. The publication period was from inception to 5 June 2025, and the search terms were “Mediterranean diet AND cognitive AND children”.

### 2.3. Study Selection

Following the removal of the duplicates, two authors independently screened the titles and abstracts to identify potentially eligible studies for inclusion in this systematic review. Full texts deemed potentially relevant by both authors were independently assessed according to the eligibility criteria. Excluded studies, along with the reasons for their exclusion, were documented in a Microsoft Office Excel (Microsoft Corporation, Redmond, WA, USA) file. This file is provided as to ensure transparency.

### 2.4. Data Extraction and Quality Assessment

Data were extracted from the full-text articles that met the inclusion criteria using a pre-designed data extraction sheet. The extracted data included the following: the first author’s last name, year of publication, population details (number, age, sex, and country of origin of participants), exposure/comparator (dietary patterns analyzed), outcomes (dietary assessment tools, dietary patterns, cognitive function variables such as attention, memory, language, creativity, reading comprehension, etc., and their significant associations), and study type. These data were then summarized in a qualitative table.

Two investigators independently performed the data extraction and assessed the risk of bias. The Newcastle–Ottawa Scale (NOS), adapted for cross-sectional studies, was used to evaluate the quality of the included observational studies. The NOS evaluates three domains: study selection, comparability, and exposure. The overall score on the NOS ranges from 0 to 10 stars. Studies scoring < 5 stars were categorized as low quality, those scoring 5–6 stars as medium quality, and those scoring > 6 stars as high quality [23].

## 3. Results

### 3.1. Flow of Studies Through the Review

**Figure 1:**
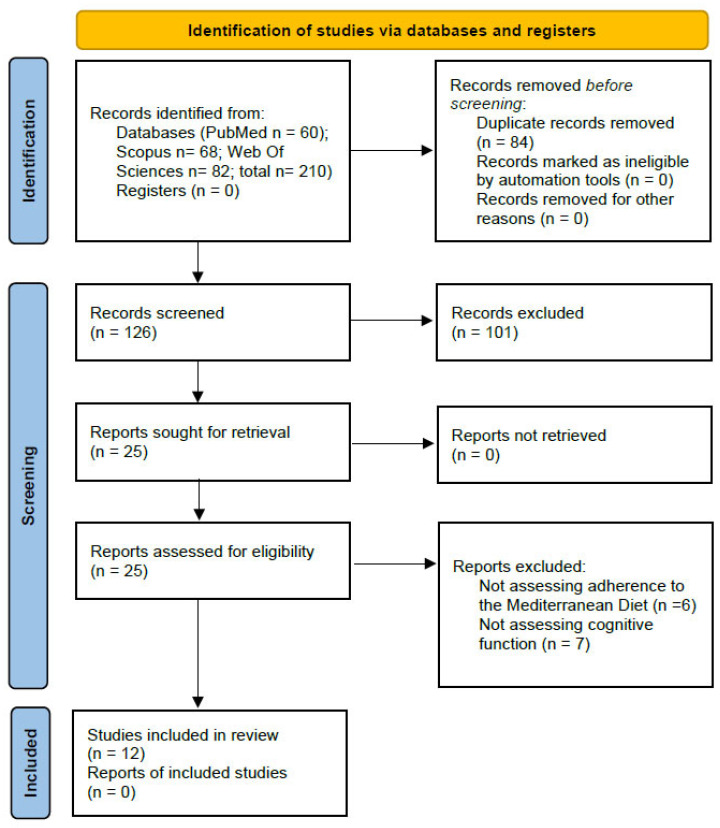
PRISMA 2020 flow diagram for new systematic reviews that included searches of databases and registers only.

Figure 1 shows the flow diagram for the included studies.

### 3.2. Search Outcome

The PRISMA flow diagram shows the detailed selection process (Figure 1). The search strategy identified a total of 210 articles from the databases. After removing duplicates, 126 articles were initially screened by title and abstract, of which 25 were identified as potentially relevant. The full-text examination further excluded 14 studies, leaving 12 studies for inclusion in this analysis, all of which were observational studies. 

### 3.3. Characteristics of the Included Studies

#### 3.3.1. Participants

Table 1 shows the main characteristics of the twelve studies of this systematic review, including a total of 6378 children and adolescents (two studies [24,25] used the same sample) aged 4–17 years (48% male). A total of eight studies focused on children, and four studies focused on adolescents. The studies were conducted in Spain [26,27,28], Italy [8,15], Chile [9,24,25,29], Greece [30], and Finland [31], and one study [32] was conducted in multiple countries (Austria, France, Germany, Greece, and Spain).

The outcomes were grouped into two different categories: dietary patterns and cognitive function.

#### 3.3.2. Adherence to the MedDiet and Dietary Patterns

When assessing adherence to the MedDiet, four studies [9,15,26,27] utilized the KIDMED Questionnaire. This questionnaire consists of 16 items that positively evaluate the consumption of fruits, vegetables, fish, whole grains, legumes, olive oil, and dairy products while negatively evaluating the consumption of fast food, sweets, and refined cereals and skipping breakfast. In the three studies conducted in countries with a MedDiet culture [15,26,27], adherence to the MedDiet was medium to high. However, in the study conducted in Chile [9], only 25.3% of participants had an adequate adherence to the MedDiet.

The other studies included in this review used various validated questionnaires to assess adherence to the MedDiet: the MedDiet score [8,31,32], the Krece Plus Questionnaire [24,25,29], the alternate MedDiet score [28], and the Food Frequency Questionnaire [30]. Most of these studies reported medium adherence to the MedDiet [8,24,25,29,30,32], while Leventakou et al. [30] did not report this data, and Haapala et al. [31] reported low adherence to this dietary pattern (study conducted in Finland).

#### 3.3.3. Cognitive Function

Regarding the evaluation of cognitive function, the studies included in this review assessed various aspects using multiple validated scales. The most frequently evaluated cognitive capacity was working memory [9,15,25,28,29,30] through different questionnaires, followed by attention and concentration capacity [24,27,29,32].

Other studies [8,9,28,30,31] assessed cognitive function using various questionnaires from a more holistic perspective, encompassing multiple cognitive domains: reasoning, reading comprehension and fluency, verbal ability, cognitive flexibility, total cognitive performance, and arithmetic skills.

Finally, Chacón-Cuberos et al. [26] assessed dimensions of motivation and learning strategies such as organizational strategies, critical thinking, and self-regulation.

#### 3.3.4. Associations Between Adherence to the MedDiet and Cognitive Function

Regarding the significant associations found between adherence to the MedDiet and cognitive function, three studies [9,15,30] reported significant positive associations between working memory and this dietary pattern. However, this association was not observed in three other studies [25,28,29].

In terms of attention capacity, significant positive associations with the MedDiet were found in the studies by Caamaño Navarrete et al. [24] and Carrillo-López et al. [27], but not in two other studies [29,32].

Global cognitive performance was evaluated in preschool children aged 4–5 years and was positively associated with adherence to the MedDiet in the studies conducted by Granziera et al. [8] and Leventakou et al. [30]. However, this association was not found in the study by O’Connor et al. [28].

In the domain of language development, Leventakou et al. [30] reported that children adhering to a MedDiet pattern exhibited higher verbal abilities compared to those following a Western diet or a snacky pattern. Haapala et al. [31] identified positive correlations between reading comprehension and MedDiet adherence in 3rd-grade children, but not 1st and 2nd grades. Conversely, O’Connor et al. [28] did not find significant associations between MedDiet adherence and verbal abilities.

Additionally, adherence to the MedDiet was positively associated with creativity in the study by Caamaño Navarrete et al. and with cognitive flexibility in the study by Peña-Jorquera et al. [9,25]. Creativity and cognitive flexibility are related functions [33].

Finally, Leventakou et al. [30] and Chacón-Cuberos et al. [26] found positive associations between adherence to the MedDiet and various domains of the executive function, including elaboration and organizational strategies, critical thinking, and self-regulation. 

**Figure 2:**
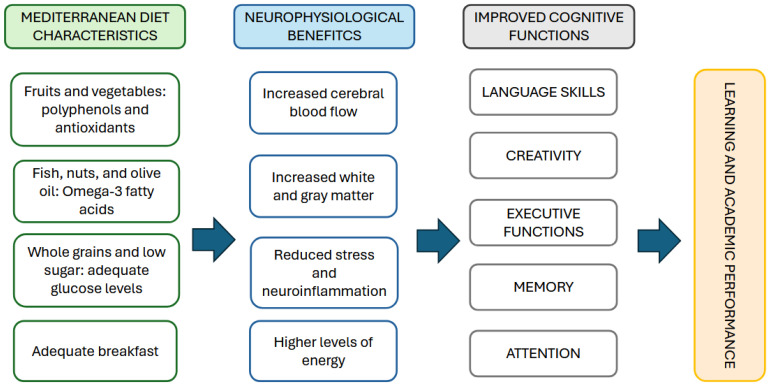
Neurophysiological positive effects of the Mediterranean diet and cognitive functions.

Figure 2 shows a summary of the main positive correlations found, and a possible physiological explanation.

### 3.4. Quality Assessment of Study Methodology

According to the NOS quality assessment, ten studies (82%) [9,15,24,25,26,28,29,30,31,32] were classified as good quality (NOS score ≥ 7) and two studies (18%) [8,27] as medium quality (4 < NOS score < 7). The most common issues identified were the lack of reporting on the non-response rate and the absence of justification for the sample size (Table 2).

## 4. Discussion

This review aimed to analyze the associations between adherence to the MedDiet and cognitive function in children and adolescents. Eleven cross-sectional studies were included, showing associations between MedDiet adherence and multiple cognitive domains, such as memory, attention, executive functions, language, and creativity. The MedDiet was beneficial for adolescents, schoolchildren, and even preschool children, highlighting the fact that healthy nutritional habits should be encouraged as soon as possible. Differences were found between studies conducted in Mediterranean countries and studies from other regions.

According to recent studies, about half of the children and adolescents in countries from the Mediterranean region have an optimal adherence to the MedDiet [34]. Accordingly, in the present review, adherence to the MedDiet was medium to high in the studies conducted in countries with a MedDiet culture [8,15,24,25,26,27,32]. However, two studies conducted in Finland [31] and Chile [9] reported low adherence to the MedDiet. As this dietary pattern may be difficult to translate to non-Mediterranean-food countries, intermediate Mediterranean-style-based patterns should be explored, studying their potential association with brain health and educational benefits [35,36].

In older adults, high adherence to the MedDiet has been associated with reduced cognitive decline and enhanced memory performance [37,38]. Additionally, multivitamin supplementation has been shown to improve memory in older adults [39], and the MedDiet is rich in various essential vitamins [4]. Moreover, healthy phospholipids abundant in the MedDiet increase cerebral blood flow and are crucial for maintaining the electrical potential of neuronal cell membranes, thereby facilitating memory formation [7]. In children and adolescents, working memory, which allows the retention of information for short periods, is essential for successfully completing assignments [40]. However, its relationship with the adherence to the MedDiet remains unexplored. In this systematic review, three studies [9,15,30] reported significant positive associations between working memory and the MedDiet. In contrast, three other studies [25,28,29] did not observe this association. A possible explanation about this association in children and adolescents may lie in the importance of avoiding prolonged fasting and consuming five daily meals, as proposed in the MedDiet, to maintain blood glucose levels and improve memory capacity [41].

In the same line, the attention capacity showed significant associations with adherence to the MedDiet in two studies [24,27] conducted on children aged 10–12 years. To explore potential physiological explanations, the questionnaires used included assessments of breakfast quality, which supports that children have the necessary energy to complete academic tasks and mitigate discomfort factors such as hunger or fatigue [42]. Although two studies [29,32] did not find significant associations between attention and MedDiet, in the study by Henriksson et al. [32], the *p*-value was 0.058. Moreover, a high-quality diet index showed a positive association with attention, and this dietary pattern shared some similarities with the traditional MedDiet, including high consumption of fruits, vegetables, fish, and whole grains.

Global cognitive performance was assessed among preschool children aged 4–5 years. Previous research has found that healthy dietary patterns favor cognitive development, but usually in older children. At the age of four, children may be too young for life-related neurodevelopment changes to manifest [28]. However, in the present review, two studies found positive associations between adherence to the MedDiet and cognitive performance in children aged 4–5 years [8,30], highlighting the relevance of promoting a healthy lifestyle as early as possible. Specifically, the high number of polyphenols present in the MedDiet could contribute to enhanced cognitive function [5,6].

Regarding the language domain, Leventakou et al. [30] reported that 4-year-old children adhering to a MedDiet pattern showed higher verbal abilities compared to those following a Western diet or a snack-based pattern, while O’Connor et al. [28] did not find significant associations. The analysis performed by Leventakou et al. [30] compared the MedDiet to other less healthy dietary patterns, which included high sugar consumption that can negatively affect cognitive performance due to reduced perfusion in the prefrontal cortex [10], potentially explaining the differences between both studies. Haapala et al. [31] observed positive associations between reading comprehension and MedDiet adherence only in 3rd grade children, but not 1st and 2nd grades, supporting the theory that the neurodevelopment related to lifestyle may not yet manifest in younger children [28], as changes in brain morphology have been observed in children aged 8 years or older [43].

Creativity and cognitive flexibility are related cognitive functions, as creativity involves the generation of new ideas, which requires cognitive flexibility to consider different perspectives and solutions. Both are essential for innovative thinking and favorize learning processes [33]. In this review, two studies [9,25] observed that a higher adherence to the MedDiet was positively associated with creativity and cognitive flexibility in children aged 11–12 years. Some specific nutrients present in the MedDiet may explain these associations. For example, omega-3 fatty acids, specifically docosahexaenoic acid, enhance membrane fluidity and modulate synaptic plasticity. B vitamins are important for DNA synthesis and neurogenesis, and polyphenols enhance cognitive performance by protecting neurons from oxidative damage [44]. Another possible link between nutrition and these cognitive functions may derive from the gut–brain axis. Recent literature suggests that gut microbiota may play a role in cognitive performance, as some gut microorganisms have been shown in laboratory animals to play a role in early brain development [45].

Adherence to the MedDiet was positively associated with executive functions in the two studies analyzing these outcomes [26,30], which included organizational strategies, critical thinking, and self-regulation. Children following a MedDiet pattern exhibited higher scores compared to those following a Western diet pattern or a snacky pattern. Similarly, reducing the consumption of refined sugars and saturated fat is crucial, as these contribute to neuroinflammation and impair study habits and effort capacity [46]. Furthermore, MedDiet is associated with a higher perception of well-being, which reduces anxiety and enhances self-regulation capacity [47]. Self-regulation is a critical factor frequently considered by teachers in assessing student performance, as it allows proper pacing and planning of tasks within time constraints [48]. Possible explanations for these benefits include nutrients abundant in the MedDiet, such as unsaturated fatty acids and polyphenols. High intake of polyphenols and omega-3 polyunsaturated fatty acids has been associated with reduced stress and negative emotions [49,50], given that depression is linked to an inflammatory state [51].

### 4.1. Clinical Implications

This systematic review highlights the benefits of the MedDiet on cognitive function in children and adolescents, which is closely related to their learning and academic performance. Therefore, schools should conduct educational workshops for families to promote this dietary pattern at home, with a focus emphasizing its health benefits, particularly its positive impact on cognitive function.

### 4.2. Strength and Limitations

Despite its novelty and its relevant results, findings from this review should be interpreted with caution due to the observational design of all included studies, which prevents establishing causality. In addition, it was not possible to perform a meta-analysis, since there were no studies that assessed the same cognitive function outcomes and adherence to MedDiet with the same instruments. Although the quality of the studies was generally high, the potential for residual confounding remains, as most studies did not fully adjust for socioeconomic variables, parental involvement, or school environment. Selection and reporting biases may also have influenced the observed associations.

### 4.3. Future Lines of Research

Future research should conduct randomized controlled studies with different MedDiet interventions (e.g., educational workshops, school meals), assessing multiple cognitive functions in children and adolescents of different school stages. In addition, it could be interesting to consider socioeconomic status, parental education, and cultural norms regarding food access that may influence both dietary habits and cognitive development in children.

## 5. Conclusions

Adherence to the MedDiet was positively associated with multiple cognitive functions in children and adolescents from different countries. The main cognitive domains related to the MedDiet were creativity, language skills, and executive functions. However, the wide variety of instruments assessing the adherence to the MedDiet and cognitive functions did not allow for performing a meta-analysis. Given the observational nature of the included studies, the results should be interpreted as associations rather than evidence of causality. Therefore, the MedDiet shows promise as a dietary pattern that may support cognitive development in youth, but further experimental research is needed to confirm these findings.

## Figures and Tables

**Table 1:** Associations between adherence to the Mediterranean diet and cognitive function in children and adolescents.

Authors	Population	Exposure/Comparator	Outcomes	Study Type
Masini 2023 [15]	n = 106 children aged 7.92 ± 1.40 years; 50% male; Italy	MedDiet	Adherence to the MedDiet (KIDMED) was positively associated with working memory (Wechsler Intelligence Scale for Children, WISC-IV)	Observational, cross-sectional
Granziera 2021 [8]	n = 54 children aged 5 years; 56% male Italy	MedDiet	Adherence to the MedDiet (MedDiet score) was positively associated with global cognitive score (Griffiths Mental Development Scales-Extended Revised, GMDS-ER)	Observational, cross-sectional
Peña-Jorquera 2024 [9]	n = 1296 children aged 11.9 ± 1.2 years; 50% male Chile	MedDiet	Adherence to the MedDiet (KIDMED) was positively associated with cognitive flexibility, executive functions, and working memory (Neurocognitive Performance Test, NCPT)	Observational, cross-sectional
Caamaño-Navarrete 2021 [24]	n = 248 children aged 11.70 years; 55% male Chile	MedDiet	Adherence to the MedDiet (Krece Plus Test) was positively associated with selective attention and concentration (d2 test)	Observational, cross-sectional
Caamaño-Navarrete 2021 [25]	n = 248 children aged 11.70 years; 55% male Chile	MedDiet	Adherence to the MedDiet (Krece Plus Test) was positively associated with creativity (CREA test), but not memory capacity (Ray’s Auditory Verbal Learning Test, RAVLT)	Observational, cross-sectional
O’Connor 2020 [28]	n = 1650 children aged 4 years; 51% male Spain	MedDiet	No associations between adherence to the MedDiet (alternate Mediterranean diet score, aMED) and cognitive development (McCarthy Scales of Children’s Abilities, MSCA)	Observational, cross-sectional
Leventakou 2016 [30]	n = 804 children aged 4 years; 51% male Greece	MedDiet, Western and Snacky Food diet patterns	Children with a MedDiet pattern (FFQ) showed a higher cognitive development (verbal, cognitive performance, memory, and executive functions) than children with a Western and snacky food diet pattern (McCarthy Scales of Children’s Abilities, MSCA)	Observational, cross-sectional
Henriksson 2017 [32]	n = 384 adolescents aged 14.7 ± 1.3 years; 57% female Austria, France, Germany, Greece, Spain	MedDiet	Adherence to the MedDiet (MedDiet score) was not associated with attention capacity (d2 test)	Observational, cross-sectional multicentre study
Carrillo-López 2023 [27]	n = 118 children aged 10.84 ± 1.20 years; 53% male Spain	MedDiet	Adherence to the MedDiet (KIDMED) was positively associated with attention (Perception of Similarities and Differences test, FACES-r)	Observational, cross-sectional
Haapala 2017 [31]	n = 161 children aged 7.7 ± 0.3 years; 54% male Finland	MedDiet	Adherence to the MedDiet (MedDiet score) was positively associated with reading comprehension (but not reading fluency and arithmetic skills) in Grade 3 schoolchildren (but not in Grade 1 and 2 schoolchildren)	Observational, cross-sectional
Chacón-Cuberos 2018 [26]	n = 1059 adolescents aged 15.23 ± 1.08 years; 51% female Spain	MedDiet	Adherence to the MedDiet (KIDMED) was positively associated with elaboration strategies, organizational strategies, critical thinking, self-regulation, time and study habits, self-regulation of effort, and intrinsically oriented goals (Motivation and Learning Strategies Short Form, MSLQ-SF)	Observational, cross-sectional
Caamaño-Navarrete 2025 [29]	n = 498 children aged 10–17 years; 53% female Chile	MedDiet	Adherence to the MedDiet (Krece Plus Test) was not associated with executive functions (attention, inhibition, working memory, and cognitive flexibility) (CogniFit neurocognitive assessment battery)	Observational, cross-sectional

Abbreviations: Mediterranean diet, MedDiet.

**Table 2:** Newcastle–Ottawa Scale (NOS) adapted for cross-sectional quality rating for the 12 included studies.

Cross-Sectional Studies	Selection				Comparability	Outcomes		Total Score (Maximum 9 *)
	Representativeness of Exposed Cohort	Sample Size	Non-Response Rate	Ascertainment of the Screening/Surveillance Tool		Assessment of Outcomes	Statistical Test	
Masini 2023 [15]	*	-	*	**	*	*	*	7
Granziera 2021 [8]	*	-	-	**	*	-	*	5
Peña-Jorquera 2024 [9]	*	*	*	**	*	**	*	9
Caamaño-Navarrete 2021 [24]	*	*	-	**	*	**	*	8
Caamaño-Navarrete 2021 [25]	*	*	-	**	*	**	*	8
O’Connor 2020 [28]	*	-	*	**	*	*	*	7
Leventakou 2016 [30]	*	*	*	**	*	*	*	8
Henriksson 2017 [32]	*	*	*	*	*	*	*	7
Carrillo-López 2023 [27]	-	*	-	**	*	-	*	5
Haapala 2017 [31]	*	*	*	**	*	**	*	9
Chacón-Cuberos 2018 [26]	*	*	*	**	*	*	*	8
Caamaño-Navarrete 2025 [29]	*	*	-	**	*	**	*	8

A * means that the study meets that criterion, or ** two criteria.

## Data Availability

The original contributions presented in this study are included in this article.

## References

[1] Hidalgo-Mora J.J., García-Vigara A., Sánchez-Sánchez M.L., García-Pérez M.Á., Tarín J., Cano A. (2020). The Mediterranean Diet: A Historical Perspective on Food for Health. Maturitas.

[2] Sánchez-Sánchez M.L., García-Vigara A., Hidalgo-Mora J.J., García-Pérez M.Á., Tarín J., Cano A. (2020). Mediterranean Diet and Health: A Systematic Review of Epidemiological Studies and Intervention Trials. Maturitas.

[3] Obeid C.A., Gubbels J.S., Jaalouk D., Kremers S.P.J., Oenema A. (2022). Adherence to the Mediterranean Diet among Adults in Mediterranean Countries: A Systematic Literature Review. Eur. J. Nutr..

[4] Petersson S.D., Philippou E. (2016). Mediterranean Diet, Cognitive Function, and Dementia: A Systematic Review of the Evidence. Adv. Nutr..

[5] Godos J., Zappalà G., Bernardini S., Giambini I., Bes-Rastrollo M., Martinez-Gonzalez M. (2017). Adherence to the Mediterranean Diet Is Inversely Associated with Metabolic Syndrome Occurrence: A Meta-Analysis of Observational Studies. Int. J. Food Sci. Nutr..

[6] Godos J., Grosso G., Castellano S., Galvano F., Caraci F., Ferri R. (2021). Association between Diet and Sleep Quality: A Systematic Review. Sleep. Med. Rev..

[7] Mazereeuw G., Lanctôt K.L., Chau S.A., Swardfager W., Herrmann N. (2012). Effects of ω-3 Fatty Acids on Cognitive Performance: A Meta-Analysis. Neurobiol. Aging.

[8] Granziera F., Guzzardi M.A., Iozzo P. (2021). Associations between the Mediterranean Diet Pattern and Weight Status and Cognitive Development in Preschool Children. Nutrients.

[9] Peña-Jorquera H., Martínez-Flores R., Espinoza-Puelles J.P., López-Gil J.F., Ferrari G., Zapata-Lamana R., Lofrano-Prado M.C., Landaeta-Díaz L., Cigarroa I., Durán-Agüero S. (2024). Adolescents with a Favorable Mediterranean-Style-Based Pattern Show Higher Cognitive and Academic Achievement: A Cluster Analysis-The Cogni-Action Project. Nutrients.

[10] Jastreboff A.M., Sinha R., Arora J., Giannini C., Kubat J., Malik S., Van Name M.A., Santoro N., Savoye M., Duran E.J. (2016). Altered Brain Response to Drinking Glucose and Fructose in Obese Adolescents. Diabetes.

[11] Montoya M.F., Susperreguy M.I., Dinarte L., Morrison F.J., San Martín E., Rojas-Barahona C.A., Förster C.E. (2019). Executive Function in Chilean Preschool Children: Do Short-Term Memory, Working Memory, and Response Inhibition Contribute Differentially to Early Academic Skills?. Early Child. Res. Q..

[12] Shi Y., Qu S. (2022). The Effect of Cognitive Ability on Academic Achievement: The Mediating Role of Self-Discipline and the Moderating Role of Planning. Front. Psychol..

[13] Tikhomirova T., Malykh A., Malykh S. (2020). Predicting Academic Achievement with Cognitive Abilities: Cross-Sectional Study across School Education. Behav. Sci..

[14] García-Hermoso A., Esteban-Cornejo I., Olloquequi J., Ramírez-Vélez R. (2017). Cardiorespiratory Fitness and Muscular Strength as Mediators of the Influence of Fatness on Academic Achievement. J. Pediatr..

[15] Masini A., Sanmarchi F., Kawalec A., Esposito F., Scrimaglia S., Tessari A., Scheier L.M., Sacchetti R., Dallolio L. (2023). Mediterranean Diet, Physical Activity, and Family Characteristics Associated with Cognitive Performance in Italian Primary School Children: Analysis of the I-MOVE Project. Eur. J. Pediatr..

[16] Kastner J.W., May W., Hildman L. (2001). Relationship between Language Skills and Academic Achievement in First Grade. Percept. Mot. Skills.

[17] Gajda A., Karwowski M., Beghetto R.A. (2017). Creativity and Academic Achievement: A Meta-Analysis. J. Educ. Psychol..

[18] Best J.R., Miller P.H., Naglieri J.A. (2011). Relations between Executive Function and Academic Achievement from Ages 5 to 17 in a Large, Representative National Sample. Learn. Individ. Differ..

[19] Munro B.A., Weyandt L.L., Marraccini M.E., Oster D.R. (2017). The Relationship between Nonmedical Use of Prescription Stimulants, Executive Functioning and Academic Outcomes. Addict. Behav..

[20] Lima R.A., Soares F.C., van Poppel M., Savinainen S., Mäntyselkä A., Haapala E.A., Lakka T. (2022). Determinants of Cognitive Performance in Children and Adolescents: A Populational Longitudinal Study. Int. J. Environ. Res. Public Health.

[21] López-Gil J.F., Victoria-Montesinos D., García-Hermoso A. (2024). Is Higher Adherence to the Mediterranean Diet Associated with Greater Academic Performance in Children and Adolescents? A Systematic Review and Meta-Analysis. Clin. Nutr..

[22] Page M.J., McKenzie J.E., Bossuyt P.M., Boutron I., Hoffmann T.C., Mulrow C.D., Shamseer L., Tetzlaff J.M., Akl E.A., Brennan S.E. (2021). The PRISMA 2020 Statement: An Updated Guideline for Reporting Systematic Reviews. BMJ.

[23] Stang A. (2010). Critical Evaluation of the Newcastle-Ottawa Scale for the Assessment of the Quality of Nonrandomized Studies in Meta-Analyses. Eur. J. Epidemiol..

[24] Caamaño-Navarrete F., Latorre-Román P.Á., Párraga-Montilla J., Jerez-Mayorga D., Delgado-Floody P. (2021). Selective Attention and Concentration Are Related to Lifestyle in Chilean Schoolchildren. Children.

[25] Caamaño-navarrete F., Latorre-román P., Párraga-montilla J.A., Álvarez C., Delgado-floody P. (2021). Association between Creativity and Memory with Cardiorespiratory Fitness and Lifestyle among Chilean Schoolchildren. Nutrients.

[26] Chacón-Cuberos R., Zurita-Ortega F., Martínez-Martínez A., Olmedo-Moreno E.M., Castro-Sánchez M. (2018). Adherence to the Mediterranean Diet Is Related to Healthy Habits, Learning Processes, and Academic Achievement in Adolescents: A Cross-Sectional Study. Nutrients.

[27] Carrillo-López P.J. (2023). Attention and Academic Performance: The Moderator Role of Weight Status and Diet Quality. Int. J. Instr..

[28] O’connor G., Julvez J., Fernandez-Barrés S., Navarrete-Muñoz E.M., Murcia M., Tardón A., Galán I.R., Amiano P., Ibarluzea J., Garcia-Esteban R. (2020). Association of Lifestyle Factors and Neuropsychological Development of 4-Year-Old Children. Int. J. Environ. Res. Public Health.

[29] Caamaño-Navarrete F., del-Cuerpo I., Arriagada-Hernández C., Cresp-Barria M., Hernández-Mosqueira C., Contreras-Díaz G., Valdés-Badilla P., Jerez-Mayorga D., Delgado-Floody P. (2025). Association Between Food Habits with Mental Health and Executive Function in Chilean Children and Adolescents. Children.

[30] Leventakou V., Roumeliotaki T., Sarri K., Koutra K., Kampouri M., Kyriklaki A., Vassilaki M., Kogevinas M., Chatzi L. (2016). Dietary Patterns in Early Childhood and Child Cognitive and Psychomotor Development: The Rhea Mother-Child Cohort Study in Crete. Br. J. Nutr..

[31] Haapala E.A., Eloranta A.M., Venäläinen T., Jalkanen H., Poikkeus A.M., Ahonen T., Lindi V., Lakka T.A. (2017). Diet Quality and Academic Achievement: A Prospective Study among Primary School Children. Eur. J. Nutr..

[32] Henriksson P., Cuenca-García M., Labayen I., Esteban-Cornejo I., Henriksson H., Kersting M., Vanhelst J., Widhalm K., Gottrand F., Moreno L.A. (2017). Diet Quality and Attention Capacity in European Adolescents: The Healthy Lifestyle in Europe by Nutrition in Adolescence (HELENA) Study. Br. J. Nutr..

[33] Khalil R., Godde B., Karim A.A. (2019). The Link Between Creativity, Cognition, and Creative Drives and Underlying Neural Mechanisms. Front. Neural Circuits.

[34] Bibiloni M.D.M., Gallardo-Alfaro L., Gómez S.F., Wärnberg J., Osés-Recalde M., González-Gross M., Gusi N., Aznar S., Marín-Cascales E., González-Valeiro M.A. (2022). Determinants of Adherence to the Mediterranean Diet in Spanish Children and Adolescents: The PASOS Study. Nutrients.

[35] Galbete C., Kröger J., Jannasch F., Iqbal K., Schwingshackl L., Schwedhelm C., Weikert C., Boeing H., Schulze M.B. (2018). Nordic Diet, Mediterranean Diet, and the Risk of Chronic Diseases: The EPIC-Potsdam Study. BMC Med..

[36] Feinstein L., Sabates R., Sorhaindo A., Rogers I., Herrick D., Northstone K., Emmett P. (2008). Dietary Patterns Related to Attainment in School: The Importance of Early Eating Patterns. J. Epidemiol. Community Health.

[37] Coelho-Júnior H.J., Trichopoulou A., Panza F. (2021). Cross-Sectional and Longitudinal Associations between Adherence to Mediterranean Diet with Physical Performance and Cognitive Function in Older Adults: A Systematic Review and Meta-Analysis. Ageing Res. Rev..

[38] Valls-Pedret C., Sala-Vila A., Serra-Mir M., Corella D., De La Torre R., Martínez-González M.Á., Martínez-Lapiscina E.H., Fitó M., Pérez-Heras A., Salas-Salvadó J. (2015). Mediterranean Diet and Age-Related Cognitive Decline: A Randomized Clinical Trial. JAMA Intern. Med..

[39] Yeung L.K., Alschuler D.M., Wall M., Luttmann-Gibson H., Copeland T., Hale C., Sloan R.P., Sesso H.D., Manson J.A.E., Brickman A.M. (2023). Multivitamin Supplementation Improves Memory in Older Adults: A Randomized Clinical Trial. Am. J. Clin. Nutr..

[40] Lan X., Legare C.H., Ponitz C.C., Li S., Morrison F.J. (2011). Investigating the Links between the Subcomponents of Executive Function and Academic Achievement: A Cross-Cultural Analysis of Chinese and American Preschoolers. J. Exp. Child Psychol..

[41] Peña-Jorquera H., Campos-Núñez V., Sadarangani K.P., Ferrari G., Jorquera-Aguilera C., Cristi-Montero C. (2021). Breakfast: A Crucial Meal for Adolescents’ Cognitive Performance According to Their Nutritional Status. The Cogni-Action Project. Nutrients.

[42] Jirout J., LoCasale-Crouch J., Turnbull K., Gu Y., Cubides M., Garzione S., Evans T.M., Weltman A.L., Kranz S. (2019). How Lifestyle Factors Affect Cognitive and Executive Function and the Ability to Learn in Children. Nutrients.

[43] Mou Y., Blok E., Barroso M., Jansen P.W., White T., Voortman T. (2023). Dietary Patterns, Brain Morphology and Cognitive Performance in Children: Results from a Prospective Population-Based Study. Eur. J. Epidemiol..

[44] Norris S.A., Frongillo E.A., Black M.M., Dong Y., Fall C., Lampl M., Liese A.D., Naguib M., Prentice A., Rochat T. (2022). Nutrition in Adolescent Growth and Development. Lancet.

[45] Mayer E.A., Nance K., Chen S. (2022). The Gut-Brain Axis. Annu. Rev. Med..

[46] Beilharz J.E., Maniam J., Morris M.J. (2016). Short-Term Exposure to a Diet High in Fat and Sugar, or Liquid Sugar, Selectively Impairs Hippocampal-Dependent Memory, with Differential Impacts on Inflammation. Behav. Brain Res..

[47] Gantenbein K.V., Kanaka-Gantenbein C. (2021). Mediterranean Diet as an Antioxidant: The Impact on Metabolic Health and Overall Wellbeing. Nutrients.

[48] McCloskey L.E. (2015). Mindfulness as an Intervention for Improving Academic Success among Students with Executive Functioning Disorders. Procedia Soc. Behav. Sci..

[49] Parletta N., Zarnowiecki D., Cho J., Wilson A., Bogomolova S., Villani A., Itsiopoulos C., Niyonsenga T., Blunden S., Meyer B. (2019). A Mediterranean-Style Dietary Intervention Supplemented with Fish Oil Improves Diet Quality and Mental Health in People with Depression: A Randomized Controlled Trial (HELFIMED). Nutr. Neurosci..

[50] Bayes J., Schloss J., Sibbritt D. (2020). Effects of Polyphenols in a Mediterranean Diet on Symptoms of Depression: A Systematic Literature Review. Adv. Nutr..

[51] Colasanto M., Madigan S., Korczak D.J. (2020). Depression and Inflammation among Children and Adolescents: A Meta-Analysis. J. Affect. Disord..

